# Sustainable Plastics from Biomass: Blends of Polyesters Based on 2,5-Furandicarboxylic Acid

**DOI:** 10.3390/polym12010225

**Published:** 2020-01-16

**Authors:** Niki Poulopoulou, Dimitra Smyrnioti, George N. Nikolaidis, Ilektra Tsitsimaka, Evi Christodoulou, Dimitrios N. Bikiaris, Maria Anna Charitopoulou, Dimitris S. Achilias, Maria Kapnisti, George Z. Papageorgiou

**Affiliations:** 1 Chemistry Department, University of Ioannina, P.O. box 1186, 45110 Ioannina, Greece; nikki_p@windowslive.com (N.P.); dimitrasmirnioti95@gmail.com (D.S.); geoniknikolaidis@gmail.com (G.N.N.); ilektra1204@gmail.com (I.T.); 2 Laboratory of Polymer and Dyes Chemistry and Technology, Department of Chemistry, Aristotle University of Thessaloniki, GR-54124 Thessaloniki, Greece; evicius@gmail.com (E.C.); dbic@chem.auth.gr (D.N.B.); haritop.marianna@gmail.com (M.A.C.); axilias@chem.auth.gr (D.S.A.); 3 Department of Food Science and Technology, International Hellenic University, P.O. Box 141, GR-57400 Thessaloniki, Greece; marikapn@yahoo.gr

**Keywords:** 2,5-furandicarboxylic acid, poly(ethylene 2,5-furandicarboxylate), biobased polymer, renewable resources, polymer blends

## Abstract

Intending to expand the thermo-physical properties of bio-based polymers, furan-based thermoplastic polyesters were synthesized following the melt polycondensation method. The resulting polymers, namely, poly(ethylene 2,5-furandicarboxylate) (PEF), poly(propylene 2,5-furandicarboxylate) (PPF), poly(butylene 2,5-furandicarboxylate) (PBF) and poly(1,4-cyclohexanedimethylene 2,5-furandicarboxylate) (PCHDMF) are used in blends together with various polymers of industrial importance, including poly(ethylene terephthalate) (PET), poly(ethylene 2,6-naphthalate) (PEN), poly(L-lactic acid) (PLA) and polycarbonate (PC). The blends are studied concerning their miscibility, crystallization and solid-state characteristics by using wide-angle X-ray diffractometry (WAXD), differential scanning calorimetry (DSC) and polarized light microscopy (PLM). PEF blends show in general dual glass transitions in the DSC heating traces for the melt quenched samples. Only PPF–PEF blends show a single glass transition and a single melt phase in PLM. PPF forms immiscible blends except with PEF and PBF. PBF forms miscible blends with PCHDMF and PPF, whereas all other blends show dual glass transitions in DSC and phase separation in PLM. PCHDMF–PEF and PEN–PEF blends show two glass transition temperatures, but they shift to intermediate temperature values depending on the composition, indicating some partial miscibility of the polymer pairs.

## 1. Introduction

Polymers from lignocellulosic biomass via the furan pathway, like poly(alkylene 2,5-furandicarboxylate)s consist of an exceptional class of novel materials [1,2,3,4,5]. Apart from their excellent properties and especially their gas barrier, they are characterized by a reduced carbon footprint and low nonrenewable energy consumption during their production processes [6,7].

In previous years, the biorefinery concept provided new routes for the production of biofuels, polymers and chemicals from biomass [8]. Vegetable feedstock such as sugars, vegetable oils, organic acids, glycerol, and others can be used as monomers for polymer production [9,10]. Carbohydrates and lignin are the major sources of aromatic monomers, such as 2,5-furandicarboxylic acid (FDCA), which has been screened to be one of the most important building blocks or top value-added chemicals derived from biomass by the U.S. Department of Energy [6]. Poly(ethylene 2,5-furandicarboxylate) (PEF) is the most studied furan-based thermoplastic polyester, as it is considered to be the biobased alternative to poly(ethylene terephthalate) [11,12,13,14,15]. Poly(propylene 2,5-furandicarboxylate) (PPF), poly(butylene 2,5-furandicarboxylate) (PBF) and poly(1,4-cyclohexane-dimethylene 2,5-furandicarboxylate) (PCDMF) are also very promising materials [16,17,18,19,20,21].

Apart from its renewable nature, PEF shows the most promising properties, such as very high (19-fold) reduction in CO_2_ permeability compared to PET. Regarding oxygen permeability, 10-fold reduction was also reported [22,23]. Besides, PEF exhibits mechanical and thermal properties that are comparable to those of PET, or even better; for example, its glass transition temperature is 88 °C, higher than that of PET, i.e., 80 °C [24,25,26,27,28,29,30,31].

Despite the good properties of furan-based polyesters, their industrialization might take years as there are still some drawbacks. Example giving, most of the furanoates showing slow crystallization rates have been reported in general, and only PCHDMF is fast crystallizing [32,33,34,35,36,37]. For PBF, the T_g_ is 38 °C, which is not very high [38]. Furthermore, the relatively reduced thermal stability at elevated temperatures and possible coloration during processing are also problems to face [39,40].

PEF is considered the biobased alternative to fossil-based PET. However, recent progress has enabled the production of ethylene glycol and bio-terephthalic acid, so that biobased PET is also available now [41]. Furthermore, furan-based polymers are recyclable, yet non-biodegradable [42].

Three basic ways exist to modify the polymers and thus arrive to polymeric materials with tailor-made properties. These are copolymerization, incorporation of additives resulting in composite or nanocomposite materials, or blending with other polymers. The synthesis of a series of furan-based copolymers has been reported in literature [43,44,45,46,47,48,49,50]. Furthermore, some recent works reported PEF, PPF and PBF nanocomposites [51,52,53,54,55]. The third way, that is, blending, is an easy and feasible way for modifying polymer properties. In contrast to the numerous papers on PET blends and on terephthalate polyester blends, only a few papers dealing with furan-based polyester blends have appeared, until now [56,57,58,59].

Therefore, in this work, a series of PEF and PPF blends with PEN, PLA, PC, PET as well as PBF and PCDMF were prepared. They were studied concerning their miscibility, crystallization and solid-state characteristics using wide-angle X-ray diffractometry (WAXD), differential scanning calorimetry (DSC) and polarized light microscopy (PLM). According to our knowledge, this is the first work on these blends and the aim was to explore miscibility, homogenization and thermal behavior. Based on these results, one can evaluate the potential of blending as a solution to face the main drawbacks of furan-based polymers which limit their industrialization and uses of the furanoate polyesters in industrial applications.

## 2. Materials and Methods

### 2.1. Synthesis of Polyesters

2,5-furan dicarboxylic acid (purum 97%) tetrabutyltitanate (TBT) catalyst of analytical grade and 1,4-Butanediol of analytical grade used in polyester synthesis, were purchased from Sigma-Aldrich Chemical Co. (Chemie GmbH, Steinheim, Germany). All other materials and solvents used were of analytical grade.

**Scheme 1:**
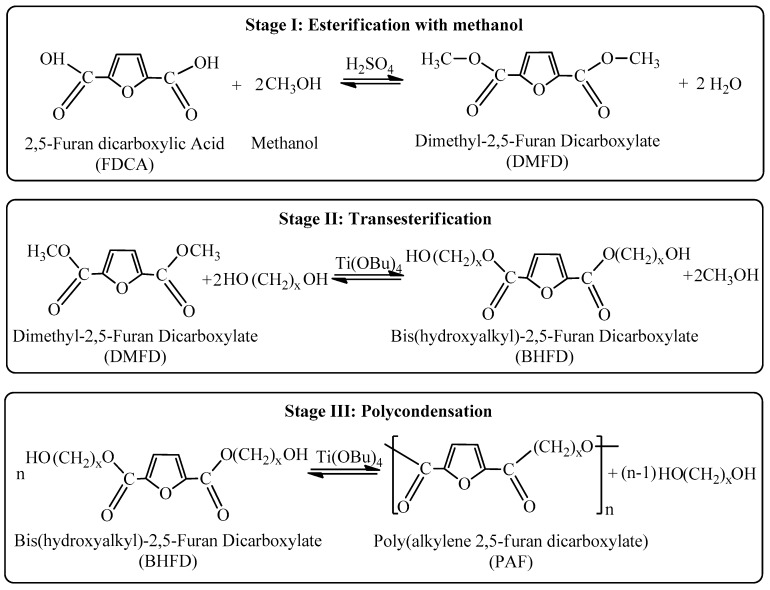
Pathway for synthesis of poly(alkylene 2,5-furandicarboxylate)s.

High molecular weight poly(ethylene 2,5-furandicarboxylate) (PEF), poly(propylene 2,5-furandicarboxylate) (PPF), poly(butylene 2,5-furandicarboxylate) (PBF) and poly(1,4-cyclohexanedimethlene 2,5-furandicarboxylate) were synthesized by applying melt polycondensation following the general procedure shown in Scheme 1 and described in detail in our previous studies [16,19,32,60]. Solid-state polycondensation (SSP) was subsequently applied to produce polymers of high molecular weight at 220 °C and 170 °C for PEF and PPF, respectively, for 8 h. PET, PPT, PBT and PBN were also prepared, as described in our previous studies, by melt polycondensation procedure [39,40]. PLA with average molecular weight Mn = 20,000 Da and polydispersity index about 1.3 and poly(bisphenol A carbonate) with average Mw about 45,000 were purchased from Sigma Aldrich Chemical Co.

### 2.2. Preparation of Polymer Blends

Polymer blends of the thermoplastic polyesters were prepared by dissolving the corresponding polymer pairs in a mixture of trifluoroacetic acid and chloroform 1/4 *v*/*v*. The solutions were poured in an excess of methanol and the blends were obtained as the precipitate. Several blends with varying compositions were prepared. Solution mixing was selected for the preparation of blends in order to avoid any possible transesterification reactions occurring at elevated temperatures during melt mixing.

### 2.3. Characterization Methods

#### 2.3.1. Intrinsic Viscosity Measurements

Intrinsic viscosity (IV) [η] measurements were performed using an Ubbelohde viscometer at 30 °C in a mixture of phenol/1,1,2,2-tetrachloroethane (60/40, *w*/*w*). IV values for neat polymers were found to be 0.63, 0.65, 0.59, 0.62, 0.62 and 0.67 dL/g for PEF, PBF, PET, PPT, PBT and PBN, respectively.

#### 2.3.2. Differential Scanning Calorimetry

The thermal behavior of the blends was studied using a Perkin Elmer Diamond DSC upgraded to DSC 8500, combined with an Intracooler IIP cooling system. Samples of about 5 mg were used. The blends were first heated at 20 °C/min up to 30 °C above the higher melting temperature and then quenched to −30 °C, before reheating at a rate of 20 °C/min, to observe the glass transition, cold-crystallization and melting of the amorphous samples. For the evaluation of the glass transition, tangents were drawn carefully on the heat flow curve at temperatures above and below the glass transition and the T_g_ was obtained as the point of intersection of the bisector of the angle between the tangents with the heat flow curve. The intersection of these tangents with that of the part corresponding to the transition were used as *T_g,onset_* and *T_g,end_*.

#### 2.3.3. X-ray Diffraction

X-ray diffraction measurements of the samples after grinding were performed using a SIEMENS Diffract 500 system employing CuKα radiation (λ = 1.5418 Å).

#### 2.3.4. Polarizing Light Microscopy (PLM)

A polarizing light microscope (Nikon, Optiphot-2) equipped with a Linkam THMS 600 heating stage, a Linkam TP 91 control unit and also a JenopticProgRes C10Plus camera were used for PLM observations.

#### 2.3.5. Fourier Transform Infrared Spectroscopy (FT-IR)

FT-IR analysis was conducted in a FT-IR spectrometer, Spectrum One by Perkin Elmer accompanied by the analogous software. Spectra were obtained over the 4000−700 cm^−1^ region and were acquired with a resolution of 4 cm^−1^ and a total of 16 scans per spectrum.

## 3. Results

Poly(alkylene 2,5-furandicarboxylate) polyester samples were synthesized following the melt polycondensation method as shown in Scheme 1. Following, a series of blends was prepared from solution, given the small amounts of the numerous samples.

For two polymers, miscibility is expected if their solubility parameter values are very close. For the polymers of this work, the solubility parameter δ values were calculated using the component group contributions (method Hoftyzer, cited byVan Krevelen [61]) and the values of the molar volumes. Given that the cohesive energy, ***E_coh_***, may be divided into three parts, corresponding with the three types of interaction forces:

(1) $$E_{coh}\;=\;E_{d}\;+\;E_{p}\;+\;E_{h}$$

with ***E_d_***, ***E_p_*** and ***E_h_*** denoting the contribution of dispersion forces, polar forces and hydrogen bonding, therefore, the solubility parameter, *δ*, can be calculated in a similar manner from the following equation:

(2) $$\delta^{2}=\;\delta_{d}^{2}+\;\delta_{p}^{2}+\;\delta_{h}^{2}$$

The solubility parameter values for the polymers of this work are summarized in Table 1.

### 3.1. PEF Blends

The first series of polyester blends were prepared by using poly(ethylene 2,5-furandicarboxylate) (PEF) as the one component combined with some of the most important industrial polyesters, including PEN, PLA, PC, etc., at several relative ratios.

**Figure 1:**
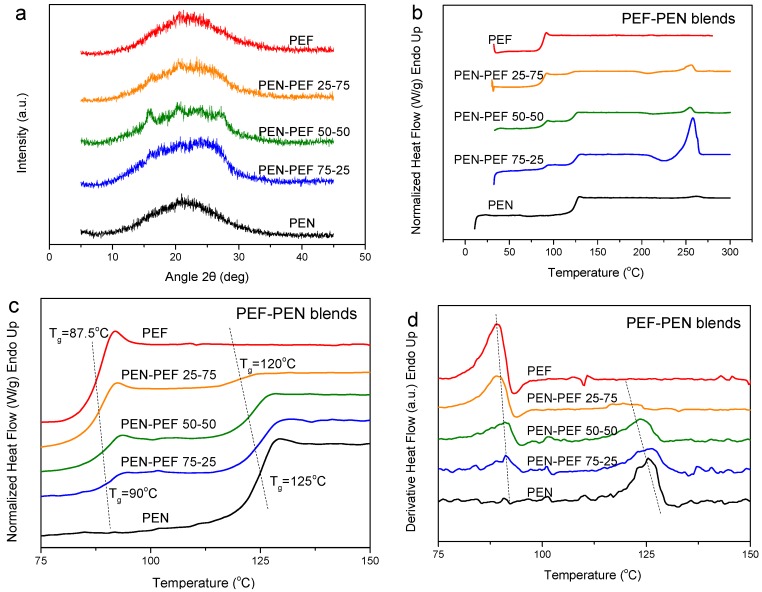
(**a**) WAXD patterns for as received PEN–PEF blends, (**b**) DSC heating thermograms, (**c**) details of DSC thermograms in the T_g_ region and (**d**) derivative heat flow for melt-quenched PEN–PEF blend samples.

PEN is one of the polyesters with high gas barrier, mechanical and thermal properties. Especially, its high glass transition temperature is of significant interest (125 °C). Therefore, PEN–PEF blends were tested. Figure 1a shows the WAXD patterns of the as prepared PEN–PEF blends. Both PEF and PEN showed negligible crystallinity and almost the same holds for their blends, judging from the very small intensity of the crystalline peaks in their respective patterns. Figure 1b shows the DSC heating scans of the blends and pure polyesters after melt-quenching. These thermograms revealed dual glass transitions. However, detailed study with the aid of the magnified traces in the temperature range of the glass transitions showed that there is some shift of the two T_g_s to intermediate values. The T_g_ for PEN shifted from 125 to 120 °C, while that of PEF from 88 to 90 °C. This trend is also clearly shown in the derivative heat flow plots against the temperature, where the glass transition appeared as peak. In conclusion, there is an indication for only partial miscibility and some homogenization in the PEN–PEF blends. This is in accordance with the solubility parameter values of Table 1, i.e., δ = 22.5 for PEF and 20.8 (MJ/m^3^)^1/2^ for PEF, which are not very close, as calculated by applying the Van Krevelen’s method.

**Figure 2:**
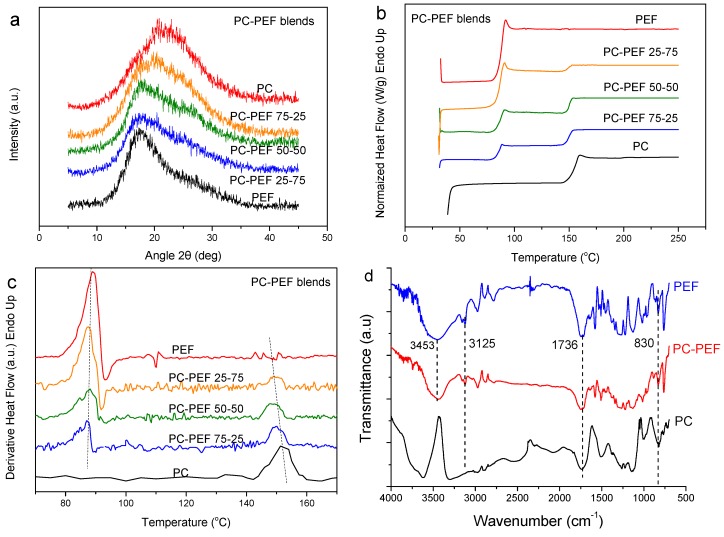
(**a**) WAXD patterns for as received PC–PEF blends, (**b**) DSC heating thermograms, and (**c**) derivative heat flow for melt-quenched PEN–PEF blend samples and (**d**) FTIR spectra of neat PC, PEF and their blend.

Another well-known high T_g_ polymer is polycarbonate. PC is an essentially amorphous polymer, as is revealed in Figure 2, and the respective WAXD pattern, for pure PC (Figure 2a). However, since PEF is also polyester of low crystallinity, the WAXD patterns of the PC–PEF blends remained amorphous, too. The DSC heating scans for the melt-quenched blend samples showed dual glass transition temperatures, and, in fact, there did not appear any real shift in the T_g_s revealing that the polymers are completely immiscible (Figure 2b,c). From FTIR measurements, the blend retains characteristics of both polyesters, meaning the C=O stretching at 1736 cm^−1^ is the angular deformation of aromatic C–H at 830 cm^−1^, terminal hydroxyl groups at 3453 cm^−1^ and the characteristic 2,5-disubstituted furan heterocycles at 3125 cm^−1^ (Figure 2d).

**Figure 3:**
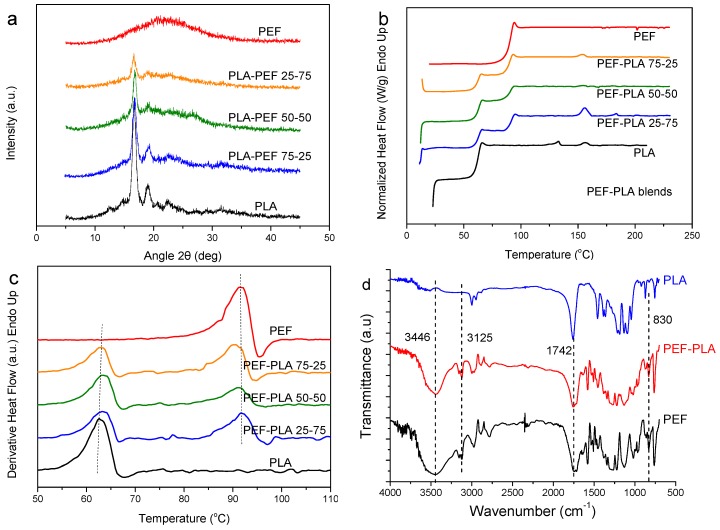
(**a**) WAXD patterns for as received PL–PEF blends, (**b**) DSC heating thermograms for melt- quenched PLA–PEF blend samples, (**c**) derivative heat flow for melt-quenched PLA–PEF blend samples and (**d**) FTIR spectra of neat PLA, PEF and their blends.

The next polymer that was blended with PEF was the biodegradable aliphatic polyester poly(L-lactic acid) (PLA). In contrast to PEF, PLA crystallized during the preparation procedure which involved solution blending and precipitation (Figure 3a). In order to study the miscibility of this polymer pair with DSC, melt-quenching was first applied. The DSC heating traces recorded for the blends after melt-quenching revealed dual glass transitions (Figure 3b). A very slight shift in the T_g_ values is not enough to indicate significant homogenization (Figure 3c). Again, from FTIR measurements, the blend retains characteristics of both polyesters, meaning that the C=O stretching at 1742 cm^−1^ (present in both neat polymers), the angular deformation of aromatic C–H at 830 cm^−1^ (present in PEF only), terminal hydroxyl groups at 3446 cm^−1^ (PEF) and the characteristic 2,5-disubstituted furan heterocycles of PEF at 3125 cm^−1^ (Figure 3d).

**Figure 4:**
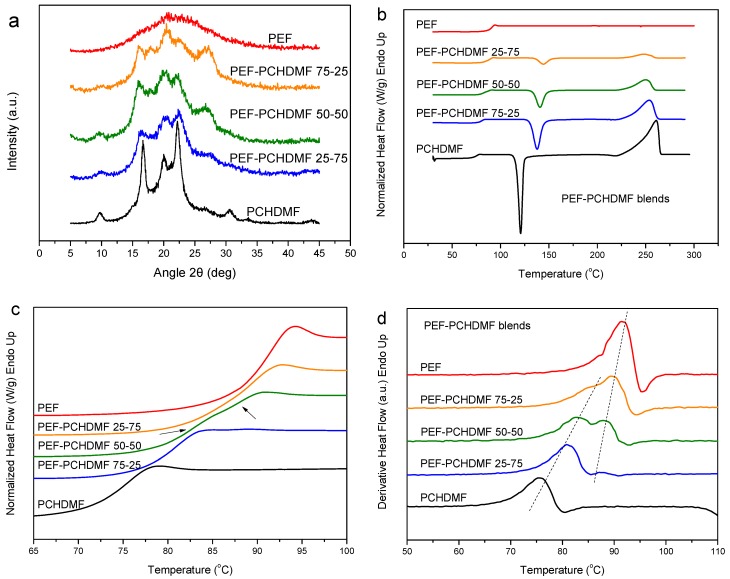
(**a**) WAXD patterns for as received PCHDMF–PEF blends, (**b**) DSC heating thermograms, (**c**) details of DSC thermograms in the T_g_ region and (**d**) derivative heat flow for melt-quenched PCHDMF–PEF blend samples.

Previous works from our group focused on the study of blends of PEF with PPF or PBF [57,58,59,62]. It was found that the PPF–PEF blends show a single glass transition. Therefore, in this work, blends of PEF with poly(1,4-cyclohexane-dimethylene 2,5-furandicarboxylate) (PCHDMF), PCHDMF–PEF, were prepared and examined to get a better insight in all furan-based blends. The as prepared blends, like PCHDMF, showed some crystallinity, basically due to the PCHDMF component (Figure 4a). The solubility parameter values for PEF [δ = 22.5 (MJ/m^3^)^1/2^] and PCHDMF [δ = 21.6 (MJ/m^3^)^1/2^] are comparable but not the same (Table 1). Of course, these values have resulted from calculations based on the Van Krevelen’s approximation and are only indicative. The DSC heating scans for the melt-quenched PCHDMF–PEF blends showed rather a single T_g_. To better study the situation, the enlarged scans in the glass transition temperature range of Figure 4c should be used or the derivative heat flow plots (Figure 4d). In both Figures, it is obvious there can be observed a shift in both T_g_s. In the derivative heat flow plot for the 50-50 composition, dual glass transitions are still observed. The picture is not very clear in the other blend compositions. On this basis, the PCHDMF–PEF blends are not completely miscible. In addition, there exists indication of some homogenization.

**Figure 5:**
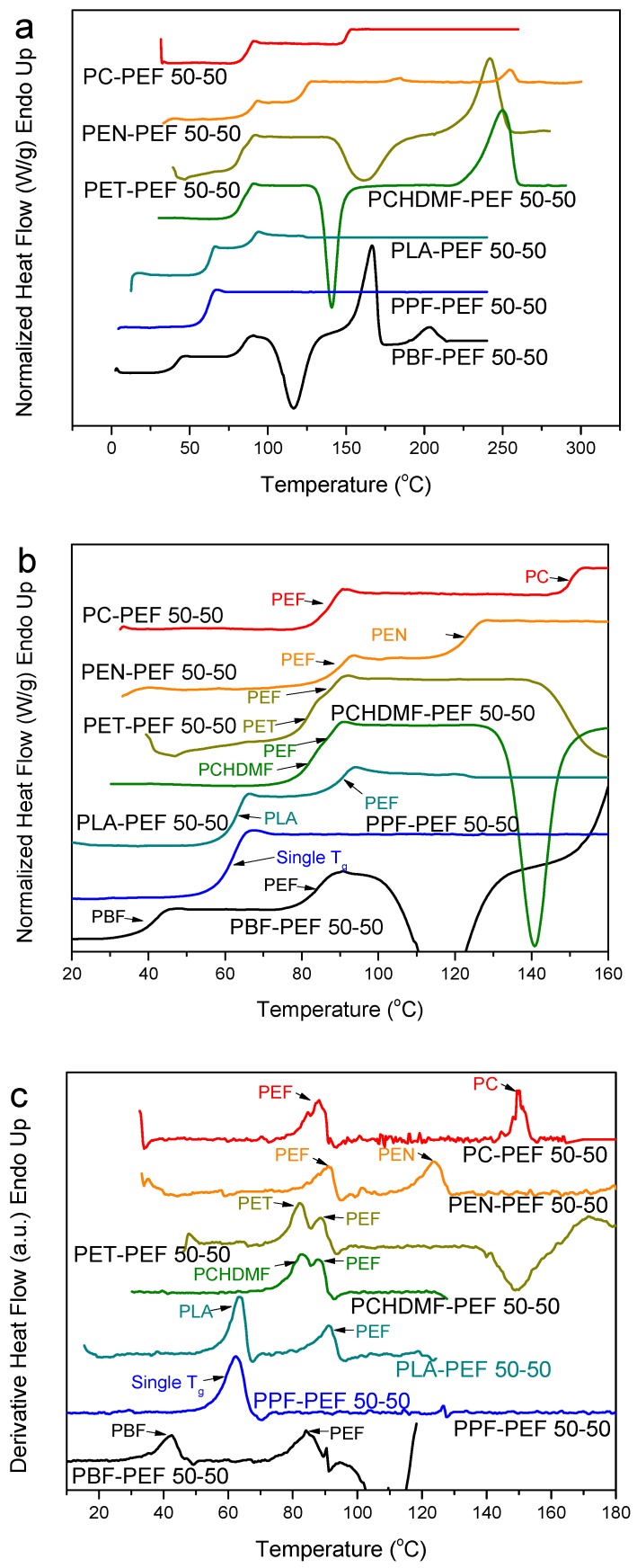
(**a**) DSC heating thermograms, (**b**) details of DSC thermograms in the T_g_ region, and (**c**) derivative heat flow for melt-quenched PEF blend samples with various polymers and 50-50 composition.

Moreover, for comparison purposes, the relative composition of PEF to some other polymers was kept constant at 50-50 and DSC heating scans for several PEF blends with PC, PEN, PET, PCHDMF, PLA, PPF and PBF are illustrated in Figure 5a. It can be seen that the PPF–PEF 50-50 blend shows a single glass transition. For the pure polymers, the T_g_s were 88 and 38 °C for PEF and PPF, respectively. For PBF–PEF 50-50 dual glass transitions were observed but with a significant shift of the two T_g_s, showing partial miscibility. Furthermore, the glass transitions are somehow resolved for PCHDMF–PEF 50-50, while the dual T_g_s are more obvious in the case of PET–PEF 50-50 in the derivative heat flow. At this point, it should be noted that the differences of the T_g_s are very small for both pairs (ΔΤ_g_ = T_g_(PEF) − T_g_(PET) = 88 − 80 = 8 °C, ΔΤ_g_ = T_g_(PEF) − T_g_(PCHDMF) = 88 − 81 = 7 °C). Using the δ parameter values, one can conclude that for PEF this value is 22.5 compared to 22 for PET and δ = 21.6 (MJ/m^3^)^1/2^ for PCHDMF. So, one would expect better homogenization in the case of PET–PEF blend. In every case, it should be noted that a very sensitive high-resolution DSC instrument was used in this work, and also the use of the derivative heat flow proved to be an important tool in resolving phenomena occurring so close in the temperature scale.

**Figure 6:**
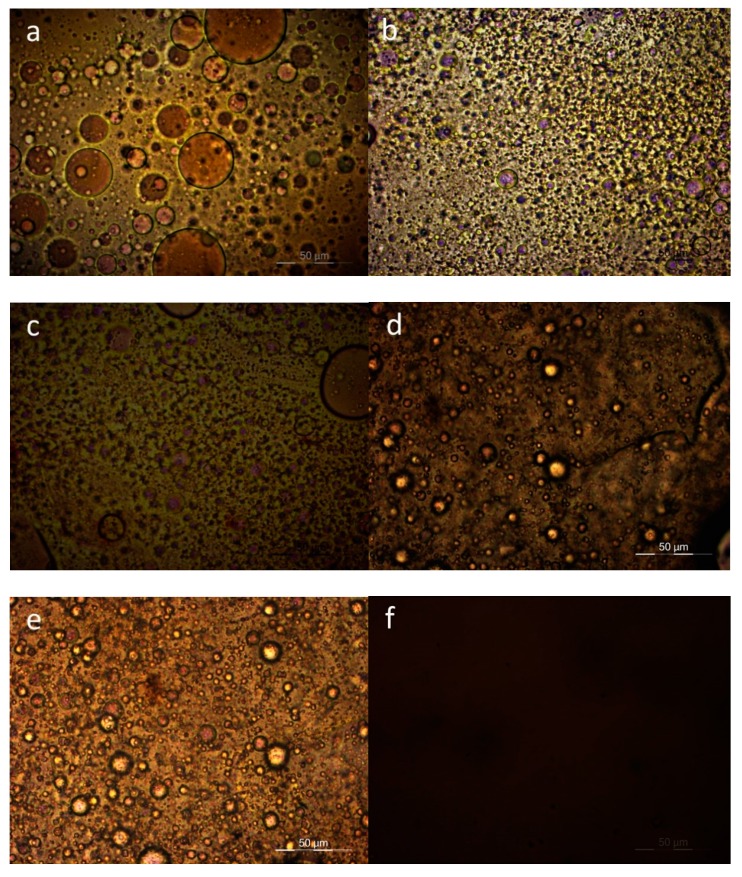

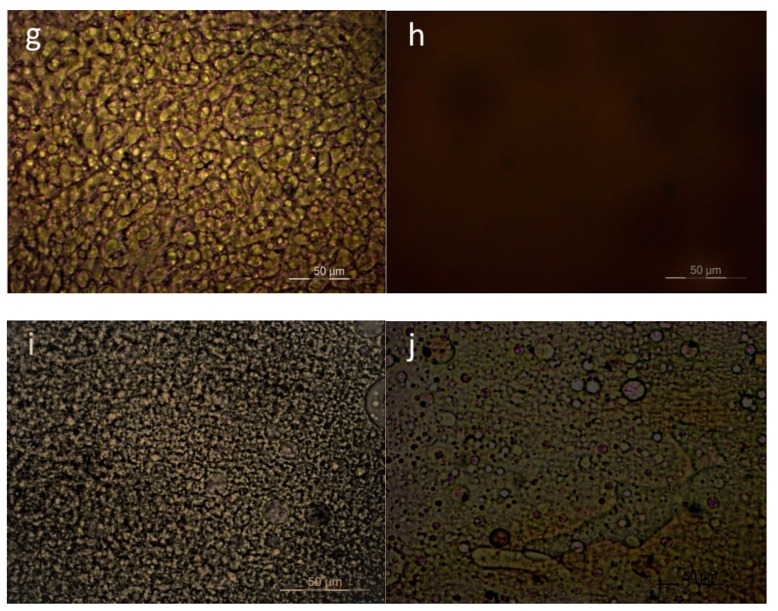
PLM photographs showing the melt phase of 50-50 blends: (**a**) PC–PEF, (**b**) PEN-–PEF, (**c**) PET–PEF, (**d**) PCHDMF-–PEF, (**e**) PLA–PEF, (**f**) PPF–PEF, (**g**) PBF–PEF, (**h**) PBF–PPF, (**i**) PPT–PPF, (**j**) PCHDMF–PPF.

**Figure 7:**
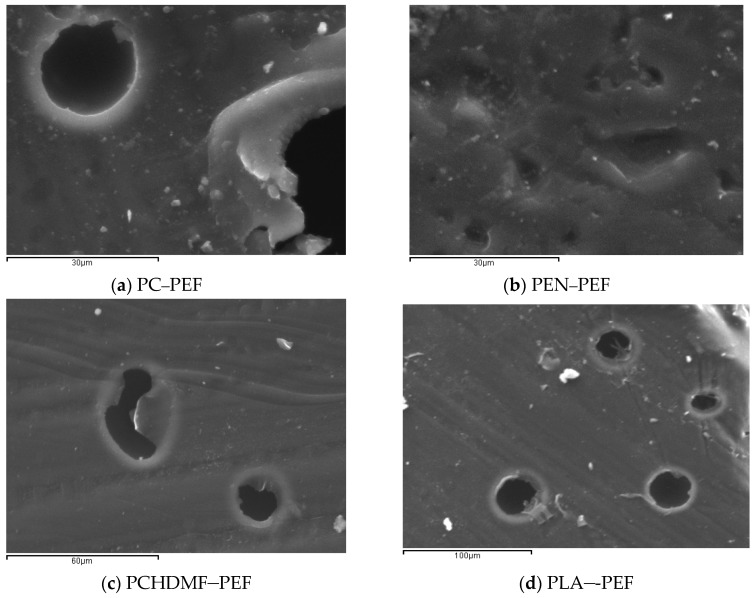

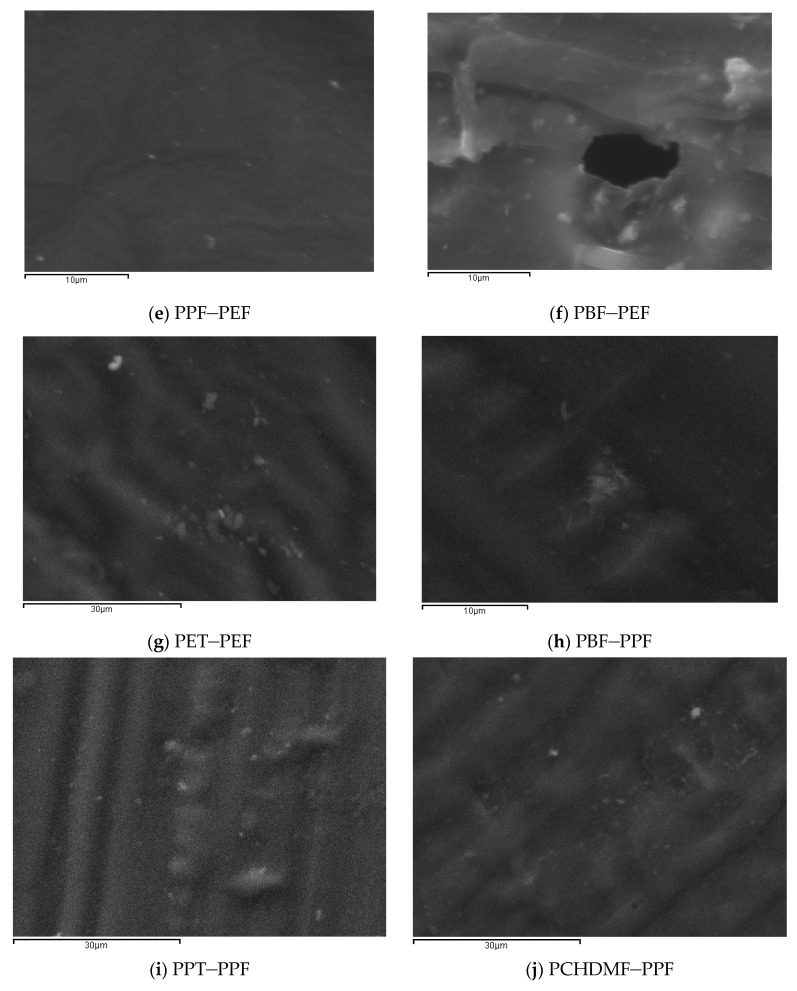
SEM photographs showing the melt-quenched samples of 50-50 blends: (**a**) PC–PEF, (**b**) PEN–PEF, (**c**) PET–PEF, (**d**) PCHDMF–PEF, (**e**) PLA–PEF, (**f)** PPF–PEF, (**g**) PBF–PEF, (**h**) PBF–PPF, (**i**) PPT–PPF, (**j**) PCHDMF–PPF.

Polarized Light Microscopy was also used for direct observations of the melt phase of the blends with 50-50 composition. The magnification was X400 in all pictures. In accordance with the DSC observations, PC–PEF 50-50 shows clear phase separation (Figure 6). PEN–PEF 50-50 shows again phase separation, despite the smaller dimensions of the separated phases. The same is observed in most cases except PPF–PEF and PBF–PPF. In the last two cases, a single melt phase was observed. Scanning Electron Microscopy (SEM) confirmed the above findings, as can be seen in Figure 7.

### 3.2. PPF and PBF Blends

**Figure 8:**
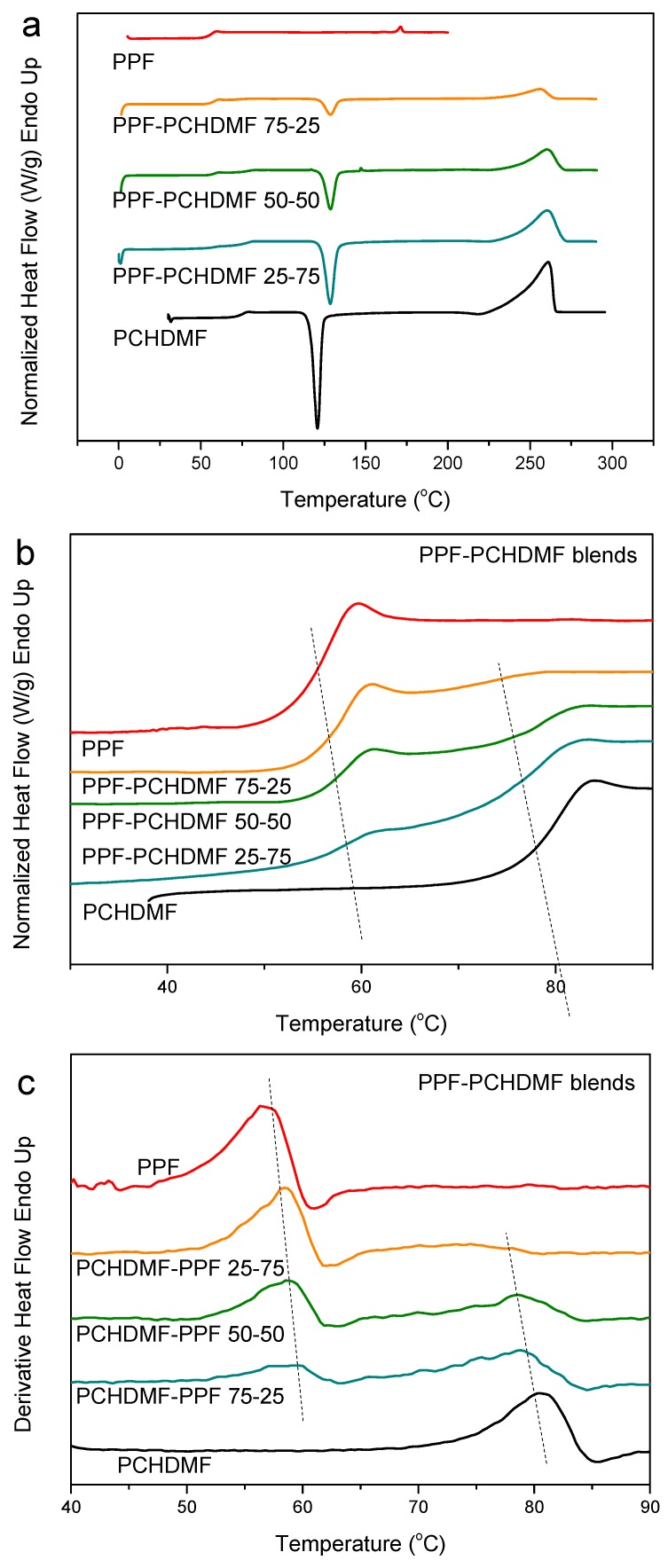
(**a**) DSC heating thermograms, (**b**) details of DSC thermograms in the T_g_ region and (**c**) derivative heat flow for melt-quenched PCHDMF–PPF blend samples.

To clarify the case and better understand the extent of miscibility and the effects of the small T_g_ difference for PCHDMF–PEF blends, the PCHDMF–PPF and PCHDMF–PBF blends were studied. In the DSC heating scans after melt-quenching, PCHDMF crystallized alone (Figure 8a). However, dual T_g_s appeared as is well-observed in the magnified scans (Figure 8b) and the derivative heat flow plots (Figure 8c). A shift, however, to intermediate temperature values can be also observed, which is a sign for partial miscibility. The T_g_ for PCHDMF decreased by about 6 °C, while the T_g_ value for PPF increased by 4 °C. It is noted that the decrease in the value of the high T_g_ is always more pronounced than the increase in the low T_g_ value in the binary blends.

**Figure 9:**
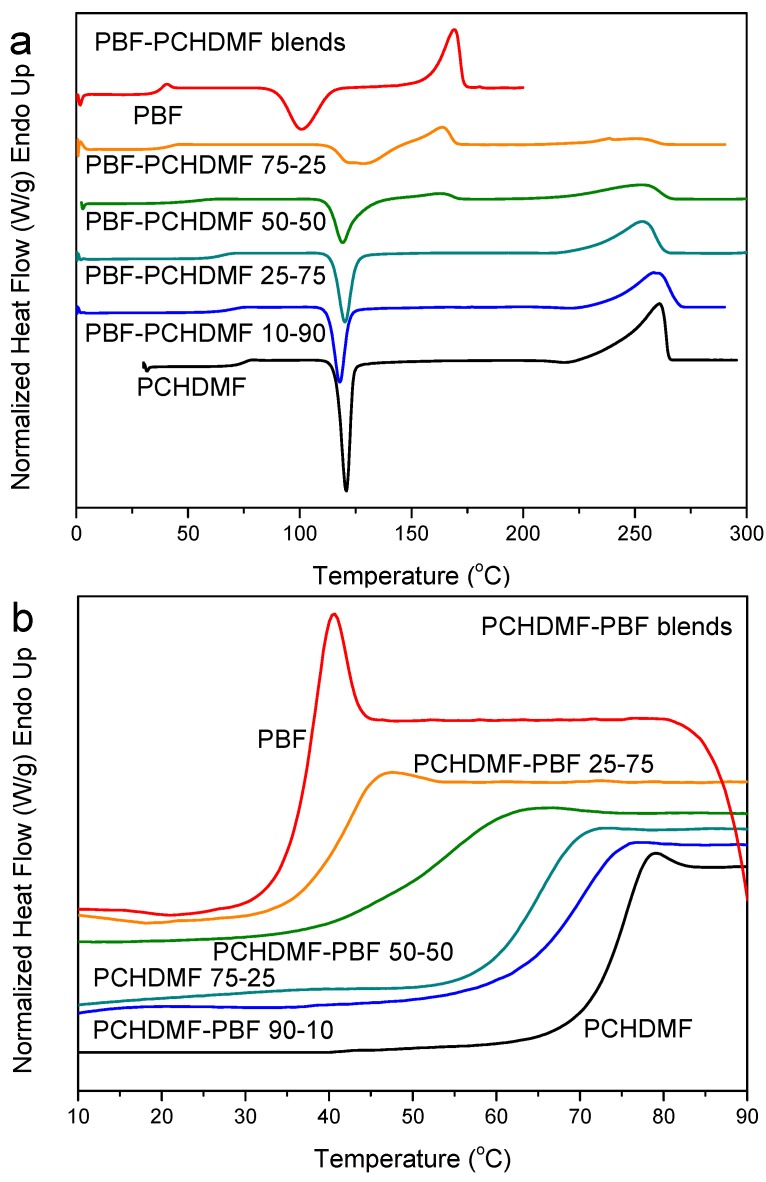

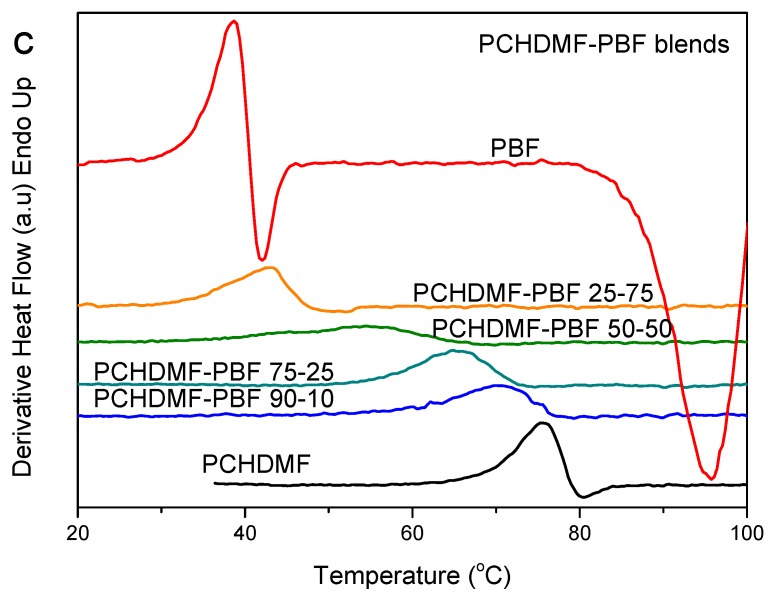
(**a**) DSC heating thermograms, (**b**) details of DSC thermograms in the T_g_ region and (**c**) derivative heat flow for melt-quenched PEN–PEF blend samples.

The PCHDMF–PBF blends also crystallized during preparation from solution. The DSC heating scans, however, for the melt-quenched blends revealed a single glass transition and also a single cold-crystallization peak at all blend compositions (Figure 9a). PBF crystallized in blends with higher than 50 wt % PBF content, that is, its crystallization was rather blocked. The same can be seen for PCHDMF. That is, the heat of fusion corresponding to its melting peak, normalized by PCHDMF mass, was a little decreased in the blends, along with a melting point depression (Figure 9a). A single composition dependent T_g_ is observed in the enlarged DSC traces of Figure 9b and the derivative heat flow curves of Figure 9c. As a matter of fact, the glass transition width was increased. Calling in mind that δ = 22.2 for PBF and 21.6 (MJ/m^3^)^1/2^ for PCHDMF, it is reasonable to suppose that PCHDMF–PBF blends can be miscible or at least partially miscible. We should note, at this point, that in a previous study PET–PBF blends were also found to show single composition dependent glass transition, while δ = 22.2 for PBF and 22 (MJ/m^3^)^1/2^ for PET [62]. PPT–PBF blends, however, (δ = 22.2 for PBF and 21.5 (MJ/m^3^)^1/2^ for PPT) showed dual glass transitions.

**Figure 10:**
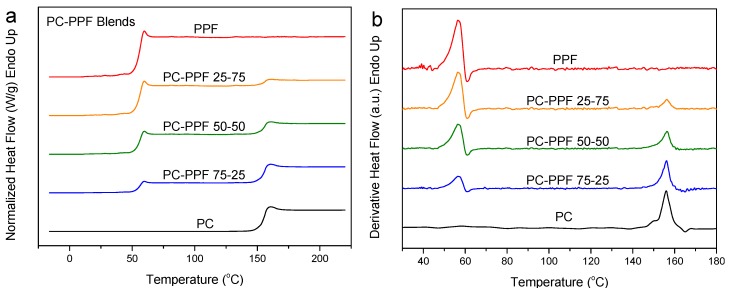
(**a**) DSC heating thermograms, and (**b**) derivative heat flow for melt-quenched PC–PPF blend samples.

Figure 10a shows the results of the DSC scans concerning the PC–PPF blends. Figure 10b shows the derivative heat flow curves against temperature. Like PEF and PBF, PPF’s mixtures with PC show two distinct T_g_s. In fact, there is no significant change in the T_g_ values of the two components in the blends. Thus, it can be assumed that PC–PPF blends are immiscible.

**Figure 11:**
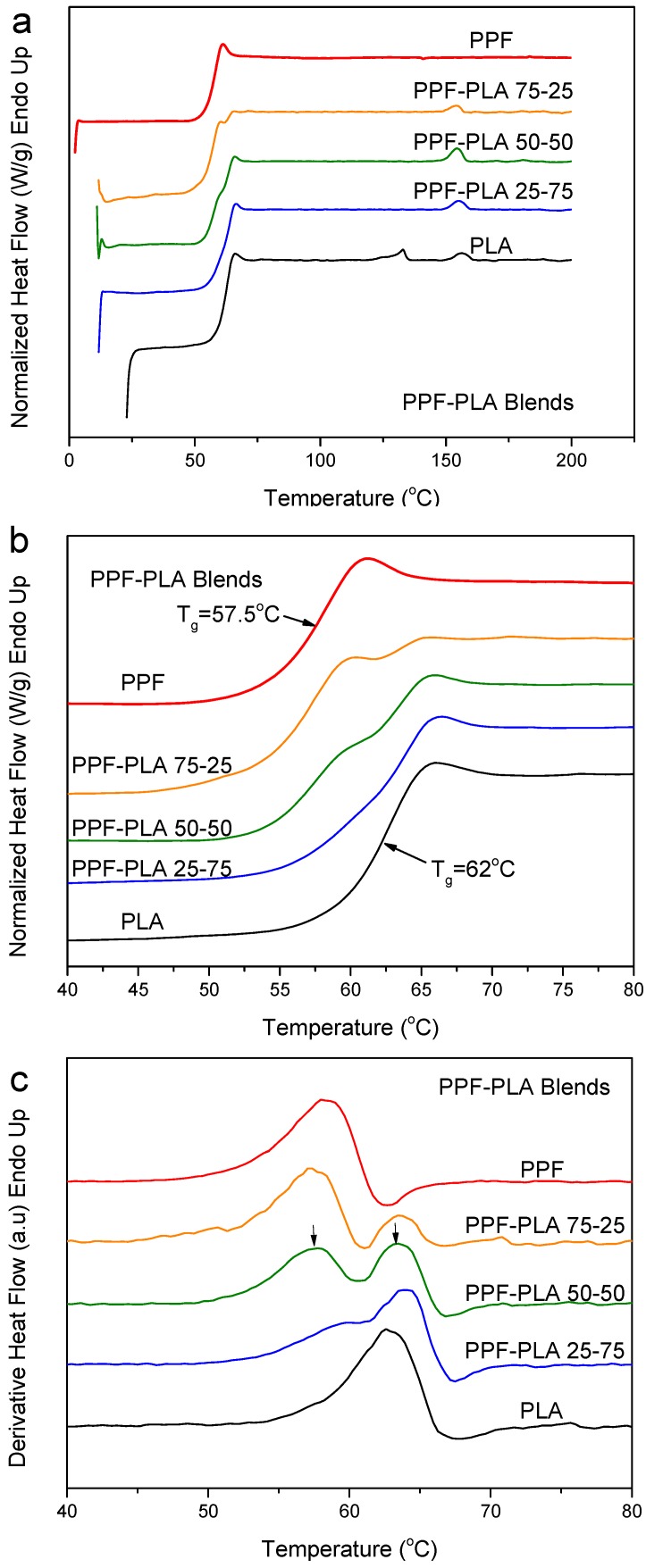
(**a**) DSC heating thermograms, (**b**) details of DSC thermograms in the T_g_ region and (**c**) derivative heat flow for melt-quenched PLA–PPF blend samples.

The case of PLA–PPF blends is studied in Figure 11. The T_g_ values for the neat polymers were 57.5 for PPF and 62 °C for PLA. So, the difference in T_g_ values is ΔT_g_ = 4.5 °C. The DSC heating traces for melt-quenched PLA–PPF blends showed two distinct glass transitions (Figure 11a). The enlarged traces of Figure 11b and the derivative heat flow curves of Figure 11c clearly show the dual processes. It was proved that even at a fast heating rate of 20 °C/min, overlapping processes are well resolved, even though some enthalpy relaxation occurred.

**Figure 12:**
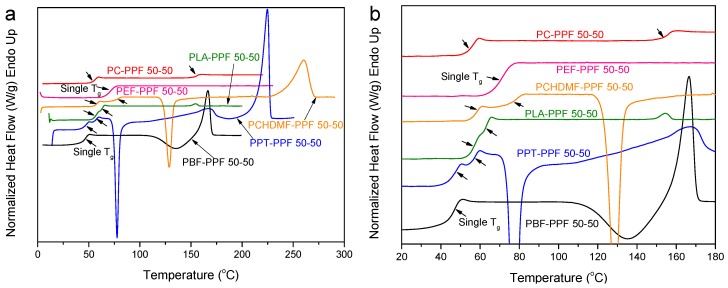
(**a**) DSC heating thermograms, (**b**) details of DSC thermograms in the T_g_ region, for melt- quenched PPF blend samples with various polymers and 50-50 composition.

To summarize the cases of PPF studied, Figure 12 shows the DSC heating scans of melt- quenched PPF blends with 50-50 composition. PPF–PEF and PBF–PPF 50-50 blends show a single composition-dependent glass transition temperature, over the whole composition range. The homogeneity of these blends was verified by the PLM study as was discussed above. The rest of the studied blends (PC–PPF, PCHDMF–PPF, PPT–PPF) showed phase separation in PLM and SEM and dual T_g_s.

**Figure 13:**
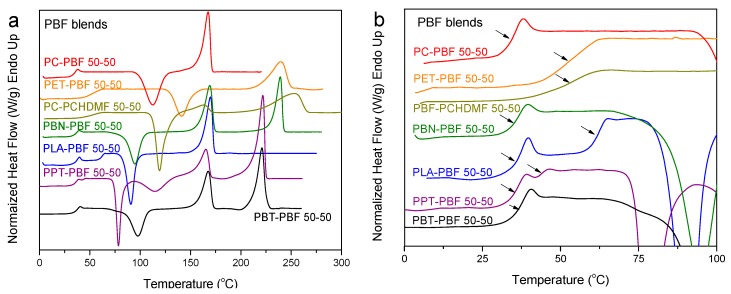
(**a**) DSC heating thermograms, (**b**) details of DSC thermograms in the T_g_ region for melt- quenched PBF blend samples with various polymers and 50-50 composition.

Finally, Figure 13 summarizes the DSC findings for quenched PBF blends. A single glass transition is observed for the PET–PBF 50-50 and PCHDMF–PBF 50-50. For PBN–PBF 50-50 and PBT–PBF 50-50, a single T_g_ can be observed; however, this is due to the fast crystallization of PBN and PBT respectively, so that the polymer pairs are phase separated due to crystallization. For PC–PBF 50-50, the glass transition for PC is just masked by the cold crystallization of PBF. In fact, PC and PBF polymers are not miscible and the T_g_ for PBF is unchanged.

## 4. Conclusions

High molecular weight poly(alkylene 2,5-furandicarboxylate) polyesters were synthesized in this work. These polymers were blended with commercially important polymers such as PET, PEN, PLA, PC, PPT. PPF–PEF blends were found to be miscible, based on DSC and PLM studies. PPF also formed miscible blends with PBF. In addition, PBF also formed miscible blends with PCHDMF and PET. All the rest of the different combinations of polymer pairs studied in this work were partially miscible. The blends with PC were essentially immiscible.

## Figures and Tables

**Table 1:** Molar volumes, V, and solubility parameter values, δ, for the polymers studied in this work and used in blends.

Polymer	V (cm^3^/mol)	δ (MJ/m^3^)^1/2^
PEF	125.7	22.5
PPF	142.1	22.4
PBF	158.4	22.2
PCHDMF	211.7	21.6
PET	144.2	22.0
PPT	160.2	21.5
PBT	196.9	21.0
PCHDMT	230.2	20.8
PEN	190.7	20.8
PPN	207.1	20.5
PBN	223.5	20.2
PCHDMN	276.7	20.2
PLA	60.7	19.9
PC	212.0	20.9
